# Seroprevalence of hepatitis B virus infection, anti-HCV antibodies and HIV and knowledge among people who use drugs attending methadone therapy clinic in Tanzania; a cross-sectional study

**DOI:** 10.1186/s12879-021-06393-0

**Published:** 2021-07-21

**Authors:** Semvua B. Kilonzo, Daniel W. Gunda, David C. Majinge, Hyasinta Jaka, Paulina M. Manyiri, Fredrick Kalokola, Grahame Mtui, Elichilia R. Shao, Fatma A. Bakshi, Alex Stephano

**Affiliations:** 1 Department of Medicine, Catholic University of Health and Allied Sciences, P.O Box 1464, Mwanza, Tanzania; 2 Department of Medicine, Bugando Medical Center, P.O Box 1370, Mwanza, Tanzania; 3 Department of Medicine, Sekou Toure Regional Referral Hospital, P.O Box 132, Mwanza, Tanzania; 4 Department of Medicine Kilimanjaro Christian University College, P.O Box 2240, Moshi, Tanzania; 5 Department of Medicine, Aga Khan Hospital, P.O Box 2289, Dar es Salaam, Tanzania

**Keywords:** Viral hepatitis, HIV, People who use drugs, Tanzania

## Abstract

**Background:**

Methadone therapy clinics have been recently introduced in Tanzania, aiming at reducing risk behaviors and infection rates of viral hepatitis and HIV among people who use drugs. The objective of this study was to estimate the prevalence, associated factors and knowledge level of these conditions among people who use drugs attending a methadone clinic in Tanzania.

**Methods:**

We enrolled 253 People who using drugs receiving Methadone therapy. Clinical data was retrospectively collected from the medical records and face-to face interviews were conducted to determine the behavioral risk factors and respondents’ knowledge on viral hepatitis and HIV.

**Results:**

An overall seroprevalence of viral hepatitis (either hepatitis B surface antigen or anti-hepatitis C virus) was 6.3%, while that of hepatitis B virus mono infection was 3.5% and anti-hepatitis C antibodies was 3.5%. Seroprevalence of HIV was 12.6%. Viral hepatitis was strongly predicted by advanced age (> 35 years) (*p* = 0.02) and staying at Kirumba area (*p* = 0.004), and HIV infection was predicted by increased age (> 37 years) (*p* = 0.04) and female sex (*p* < 0.001). Regarding the knowledge of viral hepatitis, majority of the respondents were unaware of the transmission methods and availability of hepatitis B virus vaccines and only 17% were classified as well informed (provided ≥4 correct answers out of 7 questions). Good knowledge was highly predicted by higher education level of the individual (*p* = 0.001).

**Conclusions:**

Despite the efforts to curb viral hepatitis and HIV infections through Methadone clinics, infection rates among people who use drugs are still high and the general knowledge on preventive measures is inadequate.

## Background

Viral hepatitis (VH) is an increasing global public health problem and various hepatitis viruses (A, B, C, D, and E viruses) have been implicated. While Hepatitis A (HAV) and hepatitis E (HEV) are frequent causes of acute sporadic infections and outbreaks, Hepatitis B virus (HBV) and hepatitis C virus (HCV), usually lead to chronic infections that can complicate to liver cirrhosis or hepatocellular carcinoma if un-intervened. Globally, about 257 million people are living with chronic hepatitis B (CHB) and 71 million people with hepatitis C. In sub-Saharan Africa (SSA), around 60 million people are estimated to have CHB and 10 million more to have chronic HCV infection [1]. Additionally, Africa is an epicenter for Human immunodeficiency virus (HIV), where nearly 25 million people are currently living with the disease [2].

In Tanzania, the overall seroprevalence of hepatitis B surface antigen (HBsAg), hepatitis C antibody (anti-HCV), and HIV infection is estimated to be 4.1, 2.0, and 4.7% respectively in the general community [3]. While the rate of HIV infection in the country has decreased from previous estimates [4], studies among different sub-populations suggest an escalating rate of viral hepatitis. For instance, among people who inject drugs (PWID), HBsAg seroprevalence of 7.8% has been reported recently [5], which is over 3-fold of the earlier reports in 2006 [6]. The seroprevalence of anti-HCV in this subgroup of people varies from 16.2–50.2% [5, 7, 8].

People who inject drugs are at increased risk of acquiring HIV, HBV, and HCV infections due to associated risk-related injecting behaviors and risky sexual practices. Following a surge of HIV and other blood-borne infections among PWID, the first Methadone therapy clinic (MTC) was established in Tanzania in 2011, and currently there are 6 functional clinics (three in Dar es Salaam, one in Mbeya, one in Dodoma and one in Mwanza). Mwanza clinic, where this study was done, was first opened in 2018. At present in these clinics, Methadone, which is Opioid receptor agonist, is the only used drug for the management of Opioid use disorders. However, the plans are underway to include other available approaches in the future like use of new adrenergic agonists such as Buprenorphine, use of Opioid receptor antagonists such as Naltrexone, implementation of harm reduction programs, and comprehensive HIV, Tuberculosis& hepatitis services. Around 300,000 people are estimated to be abusing narcotic drugs in the country, and among them, 30,000 use injectable drugs. Only 20% of them have been enrolled in MTCs [9]. Mwanza city, which is the second largest in the country is estimated to constitute 300 PWID [10].

The majority of studies regarding the status of this population have been based in Dar es Salaam. The aims of this study, therefore, were to estimate the seroprevalence of HBsAg, anti-HCV, and HIV and to assess the knowledge regarding viral hepatitis and HIV among PWID attending MTC at Sekou Toure Regional Referral hospital (STRRH) in Mwanza.

## Methods

### Study design and setting

This was a cross-sectional study among PWID attending MTC in Mwanza, Tanzania from September 2019 to May 2020. A convenient sampling was done, and all 256 registered participants at Sekou Toure MTC were included.

### Data collection

The included patients had attended MTC for at least 2 weeks. The patients’ files were reviewed where socio-demographic information, latest status of HBsAg, HCV antibodies, and HIV were recorded. These tests are usually done 6 monthly according to the guiding standard operating procedures [11]. Hepatitis B test was done by rapid HBsAg (Meriscreen HBsAg, Gujarat India) and hepatitis C was tested using rapid test for HCV antibody (Camp medica group, Bucharest Romania) according to the manufacturer’s instructions. HIV tests were performed according to the Tanzanian national guidelines for HIV testing [12].

A specific questionnaire was developed for this study where face-to-face interviews were conducted. Series of closed ended questions were asked regarding the types, route and duration of drug used behavioral risk factors for viral hepatitis, and HBV vaccination status. Also, seven more questions were asked regarding the respondents’ knowledge on the transmission of viral hepatitis and availability of HBV vaccine to PWID in the public health facilities. Primary outcomes were the prevalences of VH and HIV, and the knowledge level regarding HBV. Individuals were considered to have good knowledge when they answered more than 3 correct answers of seven questions.

### Data analysis

Data were computerized using Epi data version 3.1 and STATA 13 (Stata Corp LP, college station, TX) was used for analysis. Continuous variables were summarized as medians with interquartile range (IQR) and categorical variables as proportions with percentages. The comparisons between the baseline characteristics of PWID in relation to the presence of HBV, HCV or HIV were done using the non-parametric Mann Whitney U test. To determine the factors that were independently associated with HBV, HCV, and HIV infections, the simple binary logistic regression was first performed, and the variables with *p* < 0.2 were included in the multivariate model. The level of significance was set at *p* < 0.05. The goodness of fit for the final model was assessed subsequently. The sensitivity and specificity of independent factors in the final model were also assessed to determine their discriminative ability. The Receiver Operating Characteristic (ROC) curves were used according to Hanley and McNeil’s method to determine the cut points with the best sensitivity and specificity for continuous variables which were reported as proportions with 95% Confidence interval (CI).

### Ethical clearance

The permission to conduct and publish the findings from this study was sought from the Catholic University of Health And Allied Sciences (CUHAS) /Bugando Medical Centre (BMC) joint ethical committee with an ethical clearance certificate number CREC/1399/2019. The patients’ information was handled by the researcher alone and their identifiers including names and registration numbers were not included in the final analysis to further conserve confidentiality.

## Results

### Characteristics and demographics of the study participants

We recruited a total of 256 PWUD attending MTC at STRRH from September 2019 to May 2020. Three clients were excluded from the study; two did not consent and one had missing data, leaving 253 for analysis. Out of 253 PWUD, 9 were infected with HBV and 9 were positive for anti-HCV antibodies. Coexistence of HBsAg and anti-HCV antibodies was detected in 2 subjects. The seroprevalence of HIV was 32 (12.6%). Coexistence of HIV and anti-HCV was detected in 2 subjects. None of the clients was found to have HIV-HBV co infection. Demographics and characteristics of the patients with HBV infection, anti-HCV positivity, and HIV infection have been illustrated in Table 1.

**Table 1:** Baseline characteristics of PWUD in relation to the presence of HBV, anti-HCV and HIV (*n* = 253)

Variable	Total (*n* = 253)	HBV +(*n* = 9)	HBV – (*n* = 244)	*P* value	HCV + (*n* = 9)	HCV- (*n* = 244)	*P* value	HIV + (*n* = 32)	HIV – (*n* = 221)	*P* value
Sex				^a^			^a^			< 0.001
Male	230 (90.9)	9 (3.9)	221(90.6)		9(3.9)	221(90.6)		20(62.5)	210(95.0)	
Female	23(9.1)	0	23(9.4)		0	23(9.4)		12(37.5)	11(4.9)	
Age groups										
18–24	32 (12.7)	0	32(13.1)	^a^	0	32(13.1)	^a^	6(18.8)	26(11.8)	0.27
25–30	96 (37.9)	0	96(36.3)	^a^	2(22.1)	94(38.5)	0.32	3(9.4)	93(42.1)	< 0.001
31–35	51 (20.2)	3(33.3)	48(19.7)	0.32	2(22.2)	49(20.1)	0.88	7(21.9)	44(19.9)	0.79
36–40	38(15.0)	4(44.4)	34(13.9)	0.01	4(14.4)	34(13.9)	0.01	8(25.0)	30(13.6)	0.09
41–45	25(9.9)	1(11.1)	24(9.8)	0.89	1(11.1)	24(9.8)	0.89	6(18.8)	19(8.6)	0.07
46–50	9(3.6)	1(11.1)	8(3.3)	0.21	0	9(3.7)	^a^	2(6.3)	7(3.2)	0.38
> 50	2(0.8)	0	2(0.8)	^a^	0	2(0.8)	^a^	0	2(0.9)	^a^
Marital				0.51			0.95			0.02
Single	143(56.5)	4(2.8)	139(56.9)		5(55.6)	138(56.6)		12(37.5)	131(59.3)	
Married/cohabiting	110(43.5)	5(55.6)	105(43.0)	0.46	4(44.4)	106(43.4)		20(62.5)	90(40.7)	
Education				0.65			0.29			0.08
Primary or below	180 (71.2)	7(77.8)	173(70.9)		5(55.6)	175(71.7)		27(84.4)	153(69.2)	
Secondary and above	73 (28.9)	2(22.2)	71(29.1)		4(44.4)	69(28.3)		5(15.6)	68(30.8)	
Drug history										
*Types of drugs*				0.78			0.78			0.88
Opioids only	179(70.8)	6(66.7)	173(70.9)		6(66.7)	173(70.9)		23(71.9)	156(70.6)	
Opioids + other drugs	74(29.3)	3(33.3)	71(29.1)		3(33.3)	71(29.1)		9(28.1)	65(29.4)	
*Route*										
Injection only	32(12.7)	3(33.3)	29(11.9)	0.05	2(22.2)	30(12.3)	0.58	3(9.4)	29(13.1)	0.55
Snorting only	97(38.3)	0	97(39.8)	^a^	2(22.2)	95(38.9)	0.38	9(28.1)	88(39.8)	0.20
Both	124(49.0)	6(66.7)	118(48.4)	0.28	5(55.6)	119(48.8)	0.69	20(62.5)	104(47.1)	0.10
Duration of drug use										
< 5 years	39(15.4)	0	39(15.9)	^a^	0	39(15.9)	^a^	3(9.4)	36(16.3)	0.56
5–10 years	136(53.8)	4(44.4)	132(54.1)	0.57	4(44.4)	132(54.1)	0.56	15(46.9)	121(54.8)	0.40
> 10 years	78(30.8)	5(55.6)	73(29.9)	0.60	5(55.6)	73(29.9)	0.10	14(43.8)	64(28.9)	0.09

^a^ Not computed because of zero observation

People who injected drugs were predominantly males with a female: male ratio of 1:10. None of the females were found to be either positive for HBsAg or anti-HCV. Regarding the HIV infection, the females were more likely to be positive compared to their males counterparts (37.5% vs. 4.9%, *p* < 0.001). The age group of 25 to 30 years comprised the majority (37.9%) of the study population, while the elderly group (> 50 years) embraced the minority (0.8%). Significantly higher frequency of both, HBV infection and anti-HCV occurred in the age group of 36 to 40 years (*p* = 0.01 for both). Also, there was a slight trend of higher HIV infection rate towards advanced age (41–45 years), though it was not statistically significant (*p* = 0.07). In the younger population (< 30 years), occurrences of HBV infection and anti-HCV were insignificant.

HIV infection was significantly higher among the married or cohabiting PWUD (62.5% vs. 40.7%, *p* = 0.02). No statistical difference was observed between the marital status and HBV infection and anti-HCV seropositivity.

Regarding Opioid use, nearly 30% reported being using additional drugs. More than one-half of the study participants were using the injection method, and out of these, 124 (79.5%) were both injecting and snorting and 32(20.5%) were injecting only. About 30% of all drug users had used the Opioid for more than 10 years, and only 39(15.4%) had used for< 5 years. The types of drugs used, route and duration were all insignificant regarding the occurrence of HBV, HCV, or HIV. Surprisingly, none of the client have had received vaccine for HBV infection (Table 1).

### Predictors of viral hepatitis and HIV

**Fig. 1:**
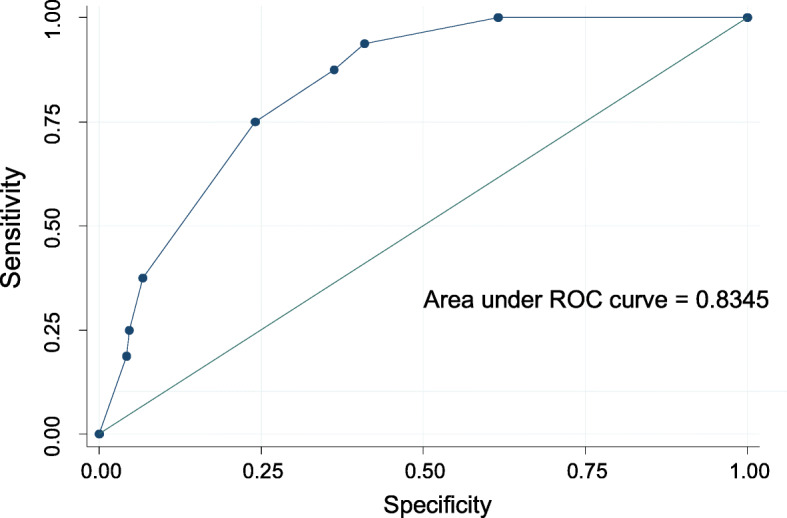
Test for goodness of fit for multivariate logistic model for viral hepatitis

**Fig. 2:**
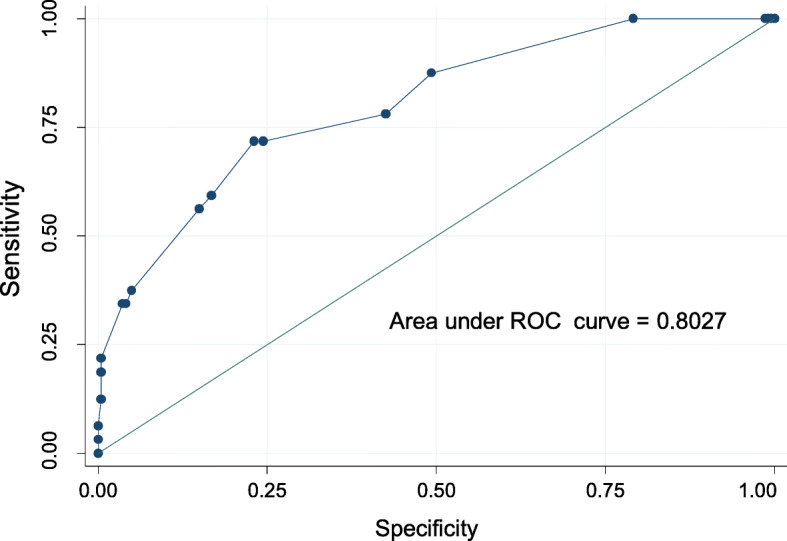
Test for goodness of fit for multivariate logistic model for HIV

Tables 2 and 3 respectively illustrate the predictors of viral hepatitis (HBsAg or anti-HCV) and HIV infection by multivariable logistic regression. Age above 35 years (*p* = 0.02) and Staying in Kirumba area (*p* = 0.004) were the only factors that were independently associated with viral hepatitis while female sex (*p* < 0.001), and age above 37 years (*p* = 0.04) strongly predicted HIV positivity. Hosmer-Lemeshow tests for goodness of fit did not indicate evidence of gross lack of fit with the area under the ROC curve of 0.83 for viral hepatitis (Fig. 1) and 0.80 for HIV (Fig. 2).

**Table 2:** Predictors of Viral Hepatitis

	HBsAg /anti-HCV positive			
	Crude Odd Ratio(95%CI)	***P*** value	Adjusted Odds Ratio (95% CI)	***P*** value
Age > 35	2.3(0.6–8.8)	0.001	4.5(1.2–16.9)	0.02
Address				
Kirumba	4.3 (1.5–11.9)	0.006	5.1(1.7–15.3)	0.004
Sahara	0.7(0.1–5.4)	0.70	α	
Bugarika	2.5(0.5–11.9)	0.27	α	
Igogo	1.1(0.1–9.4)	0.89	α	
Married (compared to single)	1.7(0.6–4.8)	0.29	α	
Low education level	10.4(0.1–2.8)	0.33	α	
Having multiple sexual partners	1.8(0.6–5.8)	0.32	α	
Using drugs> 9 years	3.8(1.2–12.0)	0.03	2.1(0.5–8.6)	0.29
Use of multiple drugs	1.2(0.3–5.0)	0.78	α	
Route of drug use, injection	3.5(1.1–10.9)	0.02	3.2(0.9–10.8)	0.06

Key: *CI* Confidence interval

^α^ Not included in multivariate analysis

**Table 3:** Predictors of HIV

	HIV			
	Crude Odds Ratio (95%CI)	***P*** value	Adjusted Odds Ratio (95%CI)	***P*** value
Female sex	11.5(4.5–29.3)	< 0.001	78.2(8.3–741.1)	< 0.001
Age > 37	3.2(1.5–6.9)	0.003	4.5(1.7–11.6)	0.002
Low education level	2.4(0.9–6.5)	0.09	1.2(0.4–3.4)	0.78
Address				
Kirumba	1.6(0.7–3.6)	0.29	α	
Igoma	1.4(0.4–6.9)	0.45	α	
Bugarika	1.1(0.2–4.9)	0.93	α	
Marital (Married)	2.4(1.1–5.2)	0.02	1.5(0.6–3.7)	0.38
Sex trade	3.7(1.3–10.5)	0.01	0.1(0.01–1.2)	0.07
Men who sex with men	1.3(0.3–6.3)	0.70	α	
Multiple sexual partners	1.6(0.7–3.5)	0.28	α	
Tatoo	0.9(0.4–2.1)	0.88	α	

Key: *CI* Confidence interval

^α^ Not included in multivariate analysis

### Knowledge of viral hepatitis

Majority of the responders didn’t know that viral hepatitis could be transmitted by sexual intercourse (92.9%), body fluids exposure (90.1%), blood exposure (84.2%), and sharing of instruments like needles (84.2%). Finally, only 51(20.1%) knew about the availability of HBV vaccines. The details have been shown in Table 4.

**Table 4:** Knowledge of PWUD about viral hepatitis (*n* = 242)

Items	***n*** = 253	%
Transmission of viral hepatitis by sexual intercourse		
YES	18	7.1
NO	235	92.9
Contaminated other body fluids as a vehicle for viral hepatitis transmission		
YES	25	9.9
NO	228	90.1
Contaminated blood as a vehicle for viral hepatitis transmission		
YES	40	15.8
NO	213	84.2
Transmission of viral hepatitis by sharing contaminated instruments, such as razors, toothbrushes or needles		
YES	40	15.8
NO	213	84.2
A neonate can get viral hepatitis infection from an infected mother		
YES	44	17.4
NO	208	82.2
Tattoo or piercing as a potential source for viral hepatitis infection		
YES	45	17.8
NO	208	82.2
Knowledge about availability of HBV vaccine to PWUD at public health facilities		
YES	51	20.1
NO	202	79.8

Forty-two, 16.6% (95%CI: 12.2–21.8) individuals were classified as well informed (4–7 correct answers) and 83.4% (95%CI: 78.2–87.8) were less informed (0–3 correct answers). Only 2, 0.8% (95%CI: 0.09–2.8) individuals responded correctly to all questions and 66.4% (95%CI: 60.6–72.6) reported incorrect answers to all questions. In multivariate logistic regression analysis, the good knowledge of viral hepatitis was found to be significantly predicted by higher education level of the individual (*p* = 0.001) (Table 5).

**Table 5:** Predictors of good knowledge about viral Hepatitis among IVDU

Variable	Knowledge Score		cOR (95%CI)	***P*** value	aOR (95%CI)	***P*** value
	Good knowledge (*n* = 42)	Poor knowledge (*n* = 211)				
Sex (Male ref. female)	39(92.8)	191(90.5)	1.4(0.4–4.8)	0.63	α	
Age (years)	31(28–37)	30(26–37)	1.0(0.9–1.1)	0.57	α	
Higher education level	26(61.9)	47(22.3)	5.7(2.8–11.4)	< 0.001	5.8(2.9–11.9)	< 0.001
HIV positive	5(11.9)	29(12.6)	0.9(0.3–2.5)	0.87	α	
HBV positive	3(7.1)	6(2.8)	2.6(0.6–10.5)	0.19	2.6(0.3–26.5)	0.42
Anti-HCV positive	2(4.8)	7(3.3)	1.4(0.3–7.3)	0.65	α	
Any viral hepatitis	5(11.9)	11(5.2)	2.4(0.8–7.5)	0.11	1.4(0.2–8.2)	0.73

Key: ^α^Not included in multivariate analysis

Good knowledge: 4–7 correct answers; Poor knowledge; 0–3 correct answers; *cOR* Crude Odds Ratio, *aOR* Adjusted Odds Ratio, *CI* Confidence interval

## Discussion

This cross-sectional study was conducted to establish the prevalence of viral hepatitis and HIV, and to determine the knowledge level among PWUD. We have reported a high seroprevalence of viral hepatitis (6.3%) and HIV (12.6%) in this population. We have also demonstrated that the occurrence of viral hepatitis is predicted by age > 35 and injection route of drug use, while that of HIV is highly predicted by age > 37 years and female sex. The general knowledge regarding to viral hepatitis is modest.

Seroprevalence of anti-HCV (3.5%) and HIV (12.6%) in this study were found to be higher than the general population in the country which is estimated to be 2 and 4.7% for HCV and HIV respectively [3, 4]. The same trend of results has been previously reported in Dar es Salaam, Tanzania where significantly higher seroprevalence of anti-HCV (57%) and HIV (40%) compared to the general population among PWUD attending MTC were reported [13]. These results support the notion that PWUD forms an important key population for HCV and HIV infections. The vast difference of rates between the later study and ours might be explained by the reasons that the later study was conducted from 2011 to 2013, immediately after commencement of MTC and harm reduction program in the country; therefore many clients were unaware of infection protection methods. Also, different study sites have different infection rates due to the urbanization effect which is a major factor for viral transmission [14–16]. This effect has further been observed in the current study where the Kirumba area, which is in the Mwanza city center, comprised significantly more cases of viral hepatitis. Regarding HBV infection, the prevalence obtained from this study (3.5%) is lower than that of the general population in the country, which is estimated to be 4.1% [3]. To date, there is no published data on the prevalence of HBV infection among PWUD enrolled in MTC. However, the magnitude of HBV infection among PWUD who are not enrolled in MTC is higher than that of the general population in the country [5, 8] and globally [17]. Therefore, our findings suggest the positive association between MTC attendance and low rates of VH in the country. Methadone replacement therapy in PWUD has been found to be effective intervention to prevent transmission of VH [18] and HIV [19], to reduce injecting-related risk behaviors [20], and to improve health-related quality of life [21], healthcare utility, utilization and expenditure [19]. Nevertheless, this promising upshot should not enervate the efforts to scale up other available modalities of management of Opioid use disorders like Opioid receptors antagonists and harm reduction that are currently inexplicit in the country, as combined prevention measures have been shown to be more effective that any measure alone [22].

We found a clear variation of age and gender profiles of PWUD and their association with VH and HIV infections. More than 90% of the studied population was males and one-half were below 30 years old. This is in line with a recent large meta-analysis review where it was reported that in low and mid-income countries PWUD were exclusively males and young adults while more aging with increasing women population was evidenced in high-income countries [17]. Furthermore, it has been previously reported that the occurrence of VH among PWUD increases with age, with no effect on gender despite the males’ preponderances [5, 7, 8], while that of HIV infection is strongly predicted by female sex [23, 24]. Similar findings have been observed in this study. Tanzania is an endemic country for HBV infection and the commonest (> 90%) mode of transmission is perinatally through mother-to-child, with the trivial horizontal transmission in the general population. Most of the people born before the year 2002 when the vaccine against HBV was introduced in the country, remain unvaccinated to date [3], and due to unavailability of HBV Immunoglobulin, children born to HBsAg positive mothers, are likely to be habitually infected. Thus, by having insignificant cases of HBV infection among the young adults that are mostly unvaccinated indicates that transmission is conspicuously horizontal among PWUD and likely driven by riskier substance use behaviors in this group of people.

To the best of our knowledge, this is the first study to describe knowledge of VH among PWUD that are enrolled in MTC in the country. The present study demonstrates significant deficits in knowledge about viral hepatitis transmission and the availability of HBV vaccine in the public health service facilities. Only 17% of the respondents were classified as well informed, and < 1% responded correctly to all questions. Furthermore, none of the clients had ever received the HBV vaccine. These findings are similar to previously reported data where lesser-educated responders and those who were negative for VH were likely to score low on VH knowledge [25, 26]. Some education (above primary school) strongly predicted good knowledge of VH in the index study while more than two-fold of respondents with good knowledge were observed among VH seropositive compared to their seronegatives counterparts. In the recent National Guidelines for Comprehensive Management of Opioid use disorder [27], it has been recommended that all PWUD attending MTC should receive comprehensive health education on the prevention of VH and HIV. Those who have been infected, should be linked immediately to the relevant care services while those who have not been infected by HBV, should be vaccinated. Despite this clear guidance, surprisingly none of the clients had ever received the HBV vaccine in the index study. HBV vaccination status among PWUD in the country has never been reported before. The previous studies regarding HBV vaccination statuses in the country mainly focused on the health care workers [28–30] which have also been reported to be unsatisfactory. Based on the previous reports [28], lack of publicity and access to HBV vaccination seems to be the major stumbling blocks to increased coverage.. These findings underscore the importance of intensifying health education service and improving access to HBV vaccination in this group. One major limitation for this study is inability to validate the information given by the clients.

## Conclusions

The current study has conclusively demonstrated that VH and HIV have substantial magnitude among PWUD attending MTC in Mwanza, Tanzania, and the rate increases significantly with age and geographical location for VH, and female gender for HIV. We have also revealed insubstantial knowledge of VH in this population with negligible uptake of the HBV vaccine. These findings which are rare in the country are crucial as they do not only provide important statistics among PWUD but also feature important policy inference on tailoring comprehensive prevention programs and early intervention strategies to the risky-youth population. Moreover, our findings inform the government and other stakeholders the discrepancies in the service provision systems that commenced nearly 10 years ago in the country. Finally, these findings might not be generalized hence they warrant a countrywide survey to all established MTCs for their efficiency. We recommend a more focused approach of different community programs dealing with PWUD to high-risk groups and close monitoring and enriching available MTCs in the country to meet the desired goals.

## Data Availability

The datasets used and/or analyzed during the current study are available from the corresponding author on reasonable request

## Abbreviations

BMC: Bugando Medical Centre
CUHAS: Catholic University of Health and Allied Sciences
HBsAg: Hepatitis B surface antigen
HBV: Hepatitis B virus
HCV: Hepatitis C virus
HEV: Hepatitis E virus
HIV: Human immunodeficiency virus
MTC: Methadone therapy clinic
PWID: People who inject drugs
PWUD: People who use drugs
STRRH: Sekou Toure Regional Referral Hospital
VH: Viral hepatitis
